# The challenges of big data biology

**DOI:** 10.7554/eLife.47381

**Published:** 2019-04-05

**Authors:** Sabina Leonelli

**Affiliations:** Department of Sociology, Philosophy and AnthropologyUniversity of ExeterExeterUnited Kingdom

**Keywords:** philosophy of science, big data, epistemology, pluralism

## Abstract

The availability of big data has the potential to transform many areas of the life sciences and usher in new ways of doing research. Here, I argue that big data biology also raises fundamental questions in the philosophy of science: for example, what is a good dataset, and how can reliable knowledge be extracted from big data? Collaborations between biologists, data scientists and philosophers of science will help us to answer these and other questions.

The life sciences have a long history of dealing with large quantities of data, and recent advances in experimental capabilities have vastly increased the amount of data that needs to be stored and analysed. The computational power available to researchers has also improved over time, but the volume and heterogeneity of the data regularly outstrip the strategies and tools available for their collection and analysis. Moreover, the volume of the data now available, especially in 'omics' fields, raises fundamental questions about the research process, such as the role of theory, the importance of context, and the purpose of know-how in data interpretation.

For example, there is widespread debate around the extent to which a scientist needs to be familiar with the protocols and instruments used to generate the data, and the relevant biology of the organisms at hand, in order to be able to interpret data. The extent to which algorithms can reliably identify causal links in data also remains a matter of contention: discovering that a specific gene pathway is frequently associated with a particular phenotypic trait is not the same as understanding why that is the case and whether the pathway is causing the trait.

Biological concepts – no matter how loosely defined – are always embedded in broader theoretical perspectives on how nature works.

There are many other questions that are of interest to philosophers of science. Does a reliance on big data change the very idea of biological discovery and what counts as biological knowledge? What role do theories play in data-intensive research and how does big data biology relate to hypothesis-driven, observational and exploratory research? How does the automation of data analysis affect the reliability of results? What is the difference between data and noise, and what are data in the first place? Biologists might think that these questions, while interesting and important, are somewhat removed from – and possibly irrelevant to – their everyday work. In this article, I aim to counter this perception by highlighting philosophical insights that can help to confront some of the key challenges related to the use of big data in biology.

## Big data biology meets biological pluralism

Biology is notoriously fragmented in its methods, goals, instruments and conceptual frameworks. Often, different groups – even within the same subfield – disagree over preferred terminology, research organisms, and experimental methods and protocols (Leonelli, 2012). As a consequence, one term may be used to refer to different processes, or different definitions may apply to the same term. This profound fragmentation, which philosophers call pluralism (Kellert et al., 2006), is reflected within the many technologies and domain-specific standards that are used to generate, store, share and analyse data (O'Malley and Soyer, 2012). Finding ways to tackle pluralism is a key challenge for big data biology.

It is easy to dismiss these difficulties as purely technical matters that can be overcome by, for example, using interoperable databases and file formats to integrate data from difference sources so that they can be used and re-used across a variety of research contexts. However, there are deeper conceptual and philosophical difficulties. Databases need to be accessed through a common ‘query’ system, and this raises the question of which terminologies should be used to classify the data and integrate them with other data, and what are the implications of such choices? The considerable labour involved in devising credible retrieval systems for biological databases speak to the difficulty of this task: this difficulty is illustrated by the lively debates over the definitions of terms such as 'pathogen' and 'metabolism' on the Gene Ontology database (The Gene Ontology Consortium, 2019).

The implications for big data biology are substantive. Far from being ‘the end of theory’, the computational mining of big data involves significant theoretical commitments. The choice and definition of keywords used to classify and retrieve data matters enormously to their subsequent interpretation. Linking diverse datasets means making decisions about the concepts through which nature is best represented and investigated. In other words, the networks of concepts associated with data in big data infrastructures should be viewed as theories: ways of seeing the biological world that guide scientific reasoning and the direction of research, which are often revised to take into account new discoveries (Leonelli et al., 2011). The quest for large-scale data integration makes it necessary for all biological disciplines to identify such theories and debate their implications for the modelling and analysis of big data (Leonelli, 2016).

We need to acknowledge that no data are 'raw' in the sense of being independent from human interpretation.

Philosophers have long discussed the theoretical significance of classification and naming practices in biology (Dupré, 2001), often in collaboration with taxonomists, and occasionally with molecular and developmental biologists. For example, researchers have attributed multiple meanings to the gene concept, which philosophers have documented and articulated as part of a broader investigation of the intellectual foundations and implications of the 'molecular bandwagon' that has dominated the last 50 years of biological research (Griffiths and Stotz, 2013; Rheinberger and Müller-Wille, 2017). These studies demonstrated that biological concepts – no matter how loosely defined – are always embedded in broader theoretical perspectives on how nature works (Callebaut, 2012).

This is not to say that big data biology is fully determined by pre-existing hypotheses. Rather, it draws on current theories and hypotheses but does not let them predetermine research outcomes (Waters, 2007). It is also important to note that, no matter which method is used to generate them, observations and measurements are always situated in a specific framework (Bogen, 2013). Irrespective of how standardised they are, the instruments used to generate those data are built to satisfy specific research agendas (Rheinberger, 2011). This means that we need to acknowledge that no data are 'raw' in the sense of being independent from human interpretation. Moreover, data can be processed differently. It is thus important to understand the conceptual choices that shaped the production and classification of data. Researchers using big data need to recognise that the theoretical structures that informed the production and processing of the data will influence their future use.

One might ask if pluralism is an obstacle to the integration of data from different sources and to the extraction of reliable and accurate knowledge from these data. Philosophers of science have argued that pluralism may actually be beneficial when attempting to extract knowledge about the highly complex and variable processes encountered in the life sciences (Dupre, 1993; Mitchell, 2003). Fragmented research traditions arise from centuries of fine-tuning research tools in order to study a given process or species in as much detail as possible. While this makes it more challenging to generalise these tools and the resulting knowledge (Levins, 1984; Wimsatt, 2007), it also ensures that the data collected are robust and inferences are accurate (Longino, 2013; Wylie, 2017). It is crucial for big data biology to build on this legacy by creating ways to work with data from diverse sources without misinterpreting their provenance or losing the insights they provide into the complexity of life.

## Assessing the quality of data

Biologists often have feelings of unease about the quality of data and metadata found in online databases, particularly when the relevant databases are not curated by experts in the specific field and/or organism. Many databases are not peer reviewed or curated, and even when they are, assessments of quality and reliability are often specific to certain fields of research and cannot easily be transferred to other research fields or other kinds of studies in the same research field (Floridi and Illari, 2014; Leonelli, 2017). The potential for loss of data quality grows the more databases become interoperable, since extensive data linkage makes it possible for unreliable data sources to pollute the overall reliability of online data collections.

This is another realm where pluralism seems to be a problem for big data biology. Does a lack of consensus on how to assess the quality of data signal a distinctive weakness of how biology can (and should) engage in big data research? One way to answer is to challenge the very understanding of the data on which this question is grounded. Thinking of data as being intrinsically good or bad – independent of context and goals of inquiry – means thinking of them as being static representations of nature that are useful because they accurately and objectively document a feature of the world at a particular time and place. This view certainly motivates the search for definitive, universal and context-independent ways of assessing which data are reliable and which are not. But it does not take into account that data are often extensively processed artefacts resulting from highly planned interactions with the world; nor does it do justice to the observation that biologists have different views of what counts as reliable data, or what counts as data in the first place (Borgman, 2015; Leonelli, 2016). Thus, what constitutes as noise for one community and/or research purpose can sometimes count as data for another (McAllister, 2011; Loettgers, 2009; Woodward, 2010).

Building on these insights, I have argued that data are 'relational': in other words, the objects that best serve as data can change depending on the standards, goals and methods used to generate, process and interpret those objects as evidence (Leonelli, 2016). This explains why assessments of data quality always relate to a specific investigation. It also accounts for the reluctance of researchers to trust data sources whose history is not clearly documented, and the related drive to collect metadata about data provenance.

Data scientists sometimes underestimate the importance of linking databases to the physical samples from which the data were originally collected (such as specimens, tissues, and cell and microbial cultures). It has been shown that access to original samples enhances data reproducibility and provides researchers with better opportunities to replicate experiments and reuse data (Dietrich et al., 2014). Access to original samples also provides a concrete point of contact between research traditions and approaches, through which differences can be identified and critically examined (Leonelli and Ankeny, 2012).

Accepting a relational view of data means moving away from generic approaches to data curation towards context-sensitive approaches that include fine-grained descriptors for the data, even though this may slow down the pace of research (Leonelli and Tempini, 2018). At the same time, recognising the local and situated nature of big data selection helps to identify what conclusions can be drawn from data analysis, and evaluate how to generalise particular inferences. This is particularly useful when assessing whether a given extrapolation can be extended from one species to another (say, from rats to humans).

There is no doubt that big data mining has a powerful heuristic function: it is often the first step in any biological inquiry, helping to define the direction and scope of research (Nickles, 2018). Big data enable biologists to spot patterns and trends more effectively, and indeed, philosophers are starting to explore how data mining can help to explore, develop and verify mechanistic hypotheses (Pietsch, 2016; Ratti, 2015; Canali, 2019). At the same time, the relational view highlights how the interpretation and reliability of inferences from big data depend on two crucial factors: first, regular confrontation with other research methods, models and approaches (Elliott et al., 2016); and second, thoughtful contextualisation of data with respect to shifts in the perspective, goals and methods of investigators (Shavit and Griesemer, 2009). Taking a relational view of data means taking seriously the value- and theory-laden history of data objects. It also promotes efforts to document that history within databases, so that future data users can assess the quality of data for themselves and according to their own standards. A case in point is a recent collaboration between a taxonomist and a philosopher on the value of ambiguity in the labels used for data in biodiversity research (Sterner and Franz, 2017).

Automated data analysis is an exciting prospect for biological discovery. Far from making human judgement unnecessary, the increasing power of computational algorithms requires a proportional increase in critical thinking. Collaboration between philosophers and biologists can foster essential reflection on which parts of data browsing and integration should be conducted with the help of algorithms, and how results should be interpreted. Collaboration between philosophers and bioinformaticians (and other types of data scientists) can promote the development of data infrastructures that adequately capture the provenance of data, and encourage users to assess the quality and relevance of data in relation to their research questions.

## Note

This Feature Article is part of the Philosophy of Biology collection.
